# Thank You Reviewers!

**DOI:** 10.1186/s40878-017-0053-3

**Published:** 2017-04-20

**Authors:** Karin Milovanovic-Hanselman

**Affiliations:** Erasmus University Rotterdam, Rotterdam, The Netherlands

## Abstract

The Board of Comparative Migration Studies would like to use this opportunity to thank our reviewers for their continued support. Their expertise and hard work as a reviewer is invaluable to us and has helped the authors in improving their manuscripts.

The reviewers’ work is extremely important for us to publish high quality papers. We would like to recognize the reviewers’ contributions by publishing their names.

Maria Abascal

Joaquin Arango

Can Aybek

Martin Bak-Jorgensen

Michaela Benson

Christophe Bertossi

Pieter Bevelander

Joachim Blatter

Caroline Bledsoe

Paolo Boccagni

Elisa Brey

Kathy Burrell

Jean Pierre Cassarino

Héctor Cebolla

Manlio Cinalli

Kathy Davis

Angeliki Dimitriadi

Henrik Emilsson

Han Entzinger

Michael Eve

Adrien Favell

Allan Findlay

Fenella Fleischmann

A. Geddes

Robbie Gilligan

Laura Gonzalez-Murphy

Elzbieta Gozdziak

Izabella Grabowska

Simon Green

Anniken Hagelund

Yossi Harpaz

Claire Healy

Jonas Hinnfors

Claus Hofhansel

Nils Holtug

Marc Hooghe

Thomas Huddleston

Tomás Jiménez

Christian Joppke

Eleonore Kofman

Holger Kolb

Arjen Leerkes

John Lie

Trine Lund Thomsen

Anna Lundberg

Blanca Garces Mascarenas

Pablo Mateos

Rahsaan Maxwell

Ines Michalowski

Liza Mugge

Pontus Odmalm

Ringo Ossewaarde

Dominic Pasura

Irene Ponzo

Rogier Reekum

Niamh Reilly

Joana Sousa Ribeiro

Christof Roos

Dumitru Sandu

Simone Scarpa

Jens Schneider

Peter Scholten

Giuseppe Sciortino

Sam Scott

Deniz Sert

Emily Skop

J. Slootjes

Andrea Spehar

Sarah Spencer

Andrei Stavila

L.E. (Rens) Tacoma

Alexandre Tandé

Sorana Toma

Kate Torkington

Anna Triandafyllidou

Mark Van Ostaijen

Floris Vermeulen

Sarah Wallace Goodman

Catherine Wihtol de Wenden

Ricard Zapata-Barrero

Karin Zelano

Malisa Zobel

